# Aerogels for Thermal Protection and Their Application in Aerospace

**DOI:** 10.3390/gels9080606

**Published:** 2023-07-26

**Authors:** Runze Jin, Zihan Zhou, Jia Liu, Baolu Shi, Ning Zhou, Xinqiao Wang, Xinlei Jia, Donghui Guo, Baosheng Xu

**Affiliations:** 1 Institute of Advanced Structure Technology, Beijing Institute of Technology, Beijing 100081, China; jrz4885905@163.com (R.J.); zhouzihan19981016@163.com (Z.Z.); 13161273311@163.com (B.S.); zhouning1995@126.com (N.Z.); 3120215942@bit.edu.cn (X.W.); jiaxinlei2009@163.com (X.J.); guodonghui2020@163.com (D.G.); 2 Beijing Key Laboratory of Lightweight Multi-Functional Composite Materials and Structures, Beijing Institute of Technology, Beijing 100081, China; 3 Beijing Spacecrafts, China Academy of Space Technology, Beijing 100191, China

**Keywords:** aerogel, thermal property, mechanical property, aerospace

## Abstract

With the continuous development of the world’s aerospace industry, countries have put forward higher requirements for thermal protection materials for aerospace vehicles. As a nano porous material with ultra-low thermal conductivity, aerogel has attracted more and more attention in the thermal insulation application of aerospace vehicles. At present, the summary of aerogel used in aerospace thermal protection applications is not comprehensive. Therefore, this paper summarizes the research status of various types of aerogels for thermal protection (oxide aerogels, organic aerogels, etc.), summarizes the hot issues in the current research of various types of aerogels for thermal protection, and puts forward suggestions for the future development of various aerogels. For oxide aerogels, it is necessary to further increase their use temperature and inhibit the sintering of high-temperature resistant components. For organic aerogels, it is necessary to focus on improving the anti-ablation, thermal insulation, and mechanical properties in long-term aerobic high-temperature environments, and on this basis, find cheap raw materials to reduce costs. For carbon aerogels, it is necessary to further explore the balanced relationship between oxidation resistance, mechanics, and thermal insulation properties of materials. The purpose of this paper is to provide a reference for the further development of more efficient and reliable aerogel materials for aerospace applications in the future.

## 1. Introduction

In recent years, great progress has been made in the research of heat-resistant materials for aerospace applications. However, many limitations are still observed in terms of their high-temperature physical and chemical stability, effective service time, and energy loss. These limitations impede the further development of new aerospace vehicles. Therefore, enhancing the extreme environmental resistance of existing thermal protection materials and exploring new thermal protection material systems are crucial in meeting the urgent needs of developing hypersonic aircraft and aerospace vehicle technologies.

As a type of porous amorphous solid material, aerogel offers notable advantages in reducing solid heat conduction and limiting thermal convection within its well-developed nanoporous network structure. Kistler first demonstrated that aerogel has a thermal conductivity of only 0.02 W/(m·K)^−1^ at an ambient temperature (25 °C), which is lower than that of static air (0.025 W/(m·K)^−1^) [1]. Heat transfer in aerogels primarily occurs through solid-phase conduction and gas-phase conduction. Regarding solid-phase heat transfer, conventional thermal insulation materials have a high solid-phase heat transfer coefficient due to the short heat transfer path and the large contact area between particles. In contrast, aerogel thermal insulation materials facilitate heat transfer along an extensive pathway with minimal particle contact area, resulting in a lower solid-phase heat conduction coefficient. Regarding gas-phase heat transfer, heat transfer occurs through molecular collisions. However, the pore size of aerogels is smaller than the average free path of gas molecules, resulting in minimal heat transfer between gases. Consequently, the gas-phase heat transfer coefficient of aerogels is markedly smaller than that of conventional macroporous insulation materials. These factors contribute to the markedly superior thermal insulation capabilities of aerogels. In addition, the radiative heat transfer mode of aerogel thermal insulation materials under high temperatures becomes important. Aerogels can absorb, reflect, and scatter infrared radiation by incorporating infrared sunscreens, further reducing thermal conductivity. The three basic heat transfer modes of typical research in Aerogel are solid heat conduction, gas heat conduction, and radiation [2]. On the basis of these properties, aerogels are often referred to as ‘super thermal insulation materials’ within the aerospace industry [3].

**Figure 1:**
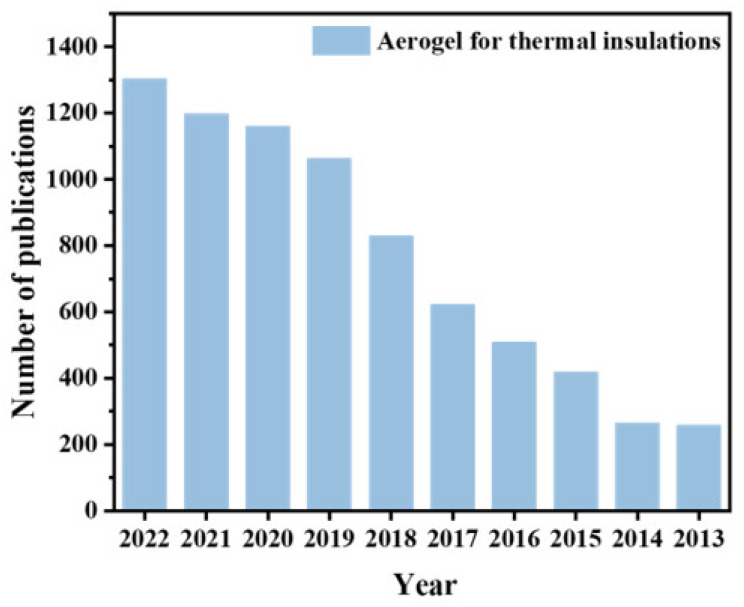
Number of articles related to thermal insulation aerogels published from 2013 to 2022 (source: Web of Science).

With the advancement of science and technology, the research focus on aerogels for aerospace thermal protection has been gradually increasing. As shown in Figure 1, the number of papers on aerogels for thermal protection has shown a consistent upward trend over the past decade. However, in recent years, there are few reports on the application status and characteristics of typical thermal protection aerogel materials, including oxide aerogels, carbon aerogels, and organic aerogels. Considering the application of aerogels in aerospace thermal protection, this paper aims to systematically review the recent progress in preparation methods, thermal insulation properties, and application status of various types of aerogels. Furthermore, it proposes a future development direction for aerogels, considering the urgent needs and research priorities in the aerospace field.

## 2. Process and Performance of the Aerogel for Thermal Protection

Since the advent of aerogels in 1931, researchers have been focused on exploring their structure and thermal protection properties [1]. In the past 90 years, remarkable advancements have been made in the development of various aerogel materials for thermal protection. On the basis of their composition and structure, aerogels can be classified into organic aerogels, inorganic oxide aerogels, and carbon aerogels. In the following sections, the research progress on different types of aerogels for thermal protection will be summarized, highlighting their compositions and characteristics.

### 2.1. Inorganic Oxide Aerogels and Composites for Thermal Protection

Inorganic oxide aerogels and composites are widely used in the aerospace industry due to their high-temperature resistance, low thermal conductivity, ease of molding, and processability. This category primarily includes single-component oxide aerogels and composites (SiO_2_, Al_2_O_3_, ZrO_2,_ etc.) and multicomponent oxide aerogels and composites (SiO_2_-Al_2_O_3_, SiO_2_-ZrO_2_, etc.).

#### 2.1.1. Single-Component Oxide Aerogels and Composites for Thermal Protection

Within the periodic table of elements, one-fifth of the elements can be employed for the preparation of single-component oxide aerogels [4]. Notably, SiO_2,_ Al_2_O_3_, and ZrO_2_ exhibit excellent thermal stability at high temperatures due to their high ionic bond energy and elevated melting points. In addition, these elements are often preferred in thermal protection due to their affordability and controllable precursors.

##### SiO_2_ Aerogel and Composites for Thermal Protection

**Figure 2:**
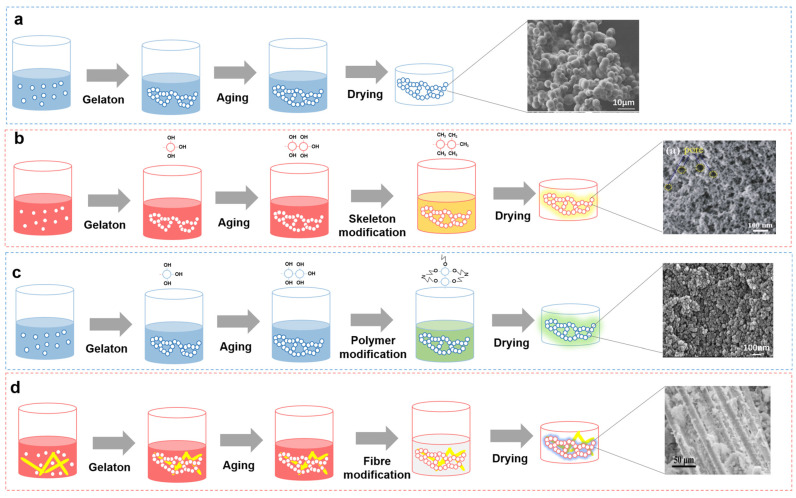
SiO_2_ aerogel prepared using the sol–gel method and various mechanical strengthening methods. (**a**) Preparation process of the traditional SiO_2_ aerogel. The right side of the figure shows a scanning electron microscopy (SEM) image of the traditional SiO_2_ aerogel. Reproduced with permission [9]. (**b**) Preparation process of the surface-modified SiO_2_ aerogel. The right side of the figure shows an SEM image of the surface-modified SiO_2_ aerogel. Reproduced with permission [6]. (**c**) Preparation process of the polymer-modified SiO_2_ aerogel. The right side of the figure shows an SEM image of the polymer-modified SiO_2_ aerogel. Reproduced with permission [7]. (**d**) Preparation process of the fiber-modified SiO_2_ aerogel. The right side of the figure shows an SEM image of the fiber-modified SiO_2_ aerogel. Reproduced with permission [8].

SiO_2_ aerogel, the earliest and most extensively studied type of aerogel, possesses remarkable characteristics, including high porosity (80–99.8%), high specific gravity (100–1400 m^2^/g), and low density (0.003–0.4 g/cm^3^). Recent research on SiO_2_ aerogels has primarily focused on atmospheric drying, mechanical enhancement, and high-temperature radiation suppression [5]. In the aerospace industry, thermal protection materials often need to withstand extreme conditions, including high temperatures and pressures. However, the weak internal structure of SiO_2_ aerogels typically results in fracture or collapse at temperatures exceeding 650 °C, potentially leading to major engineering accidents. Consequently, the aerospace community places considerable emphasis on the mechanical reinforcement of SiO_2_ aerogels. At present, mechanical reinforcement strategies for SiO_2_ aerogels usually involve in situ network skeleton reinforcement [6], polymer composite reinforcement [7], and fiber composite reinforcement [8]. The general preparation process is shown in Figure 2.

In situ network framework reinforcement primarily aims to optimize the pore structure of aerogels by controlling their composition and synthesis process, thereby enhancing their mechanical properties. This approach can be categorized into five types based on the characteristics of the preparation methods: Heat treatment [10], coprecursor preparation [11], chemical additives [9], aging [12], and surface modification [13]. Although the in situ network skeleton reinforcement method has experienced remarkable advancements in recent years, it still faces challenges, such as high cost, long preparation cycles, and environmental hazards, which hinder its industrial application in the aerospace field.

Polymer composite reinforcement involves transferring stress from the aerogel to the polymer component by forming an interpenetrating network structure, thereby improving the mechanical properties. Depending on the characteristics of the preparation method, polymer composite reinforcement can be divided into two steps, including the solution immersion polymer modification method [14], the one-step method [15], and the chemical vapor deposition polymer method [16]. However, when polymer aerogels are applied in the aerospace field, they tend to form particle aggregates at high temperatures, leading to coking and aggregation during usage, which can block the pores and reduce heat insulation capacity.

Currently, fiber composite reinforcement is considered the most effective method for reinforcing SiO_2_ aerogels in aerospace thermal protection. In terms of fiber type, the commonly used reinforced fibers include organic and inorganic fibers. Inorganic fibers are widely studied because of their higher temperature range and better mechanical reinforcement effect. The preparation methods for inorganic fibers and SiO_2_ aerogels can be classified as molding methods [17] and gel integral molding methods [18]. In addition, inorganic fibers can be categorized in terms of their composition, such as quartz, glass, ceramics (aluminum silicate), mullite, and alumina. Table 1 provides a summary of the physical properties and mechanical parameters of these representative inorganic fibers. Quartz, mullite, and other inorganic fibers can withstand temperatures exceeding 1000 °C whilst maintaining good mechanical properties. Therefore, the addition of inorganic fibers to the SiO_2_ aerogel matrix as a toughener results in a composite aerogel material with excellent mechanical and thermal properties.

**Figure 3:**
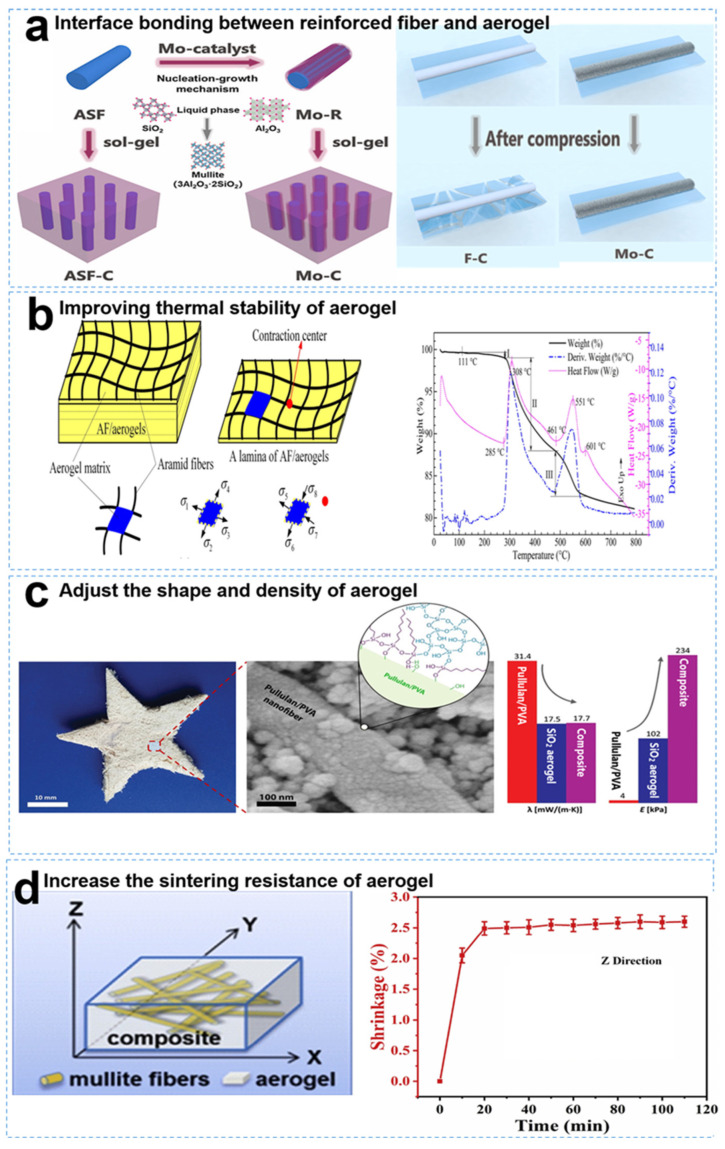
Application progress of fiber-reinforced SiO_2_ aerogel composites: (**a**) Interface bonding between reinforced fiber and aerogel. Reproduced with permission [27]. (**b**) Enhancement of the thermal stability of the aerogel. Reproduced with permission [22]. (**c**) Adjustment of the shape and density of the aerogel. Reproduced with permission [24]. (**d**) Increase in the sintering resistance of the aerogel. Reproduced with permission [26].

Current research on inorganic fiber-reinforced SiO_2_ aerogel composites for thermal protection primarily focuses on two aspects. On the one hand, SiO_2_ aerogels are tailored for specific scenarios requiring high-temperature resistance, high strength, flexibility, and other specific application requirements. On the other hand, researchers address the challenge of weak connections between micron-sized fibers and the micron-sized or even millimeter-sized gap between fibers. The first problem is primarily solved through surface modification of inorganic fibers using chemical functional groups [20], whiskers [21], and other means [22]. The second problem is primarily tackled by selecting the size [23,24] and type [25,26] of inorganic fibers. Figure 3 shows the recent research applications of fiber-reinforced SiO_2_ aerogels.

##### Al_2_O_3_ Aerogel and Composites for Thermal Protection

The aerospace industry has shown increased interest in Al_2_O_3_ aerogels for specific applications, such as hypersonic aircraft engines, due to their superior thermal stability at high temperatures (approximately 1300 °C) compared with SiO_2_ aerogels. Since the development of Al_2_O_3_ aerogels in 1975, researchers have made remarkable progress in the preparation process, performance optimization, and other aspects of thermal protective alumina aerogels.

**Figure 4:**
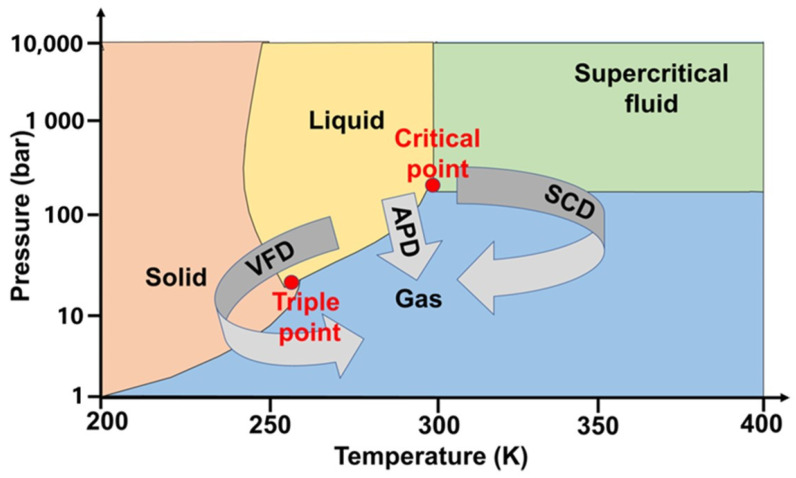
Phase diagram of different drying methods.

The preparation process of Al_2_O_3_ aerogel is similar to that of SiO_2_ aerogel, involving three steps: Wet gel preparation, gel aging, and gel drying. Depending on the precursors used, the methods can be categorized into organic alkoxide methods, inorganic salt methods, and boehmite methods. The organic alkoxide method and inorganic salt method involve the generation of Al–O–Al sol particles through the condensation reaction of the Al–OH intermediate formed after precursor hydrolysis. Subsequently, a gel is formed through a series of cross-linkages. The main difference is that the organic alkoxide method often requires the addition of chelating agents (such as ethyl acetoacetate, acetylacetone, etc. [28,29]) to form an Al–O–C structure and reduce the reaction activity because the precursor activity is typically high. The inorganic salt method involves the consumption of hydrogen ions through the ring-opening reaction of epoxides, leading to the generation and condensation of more Al–OH in the solution [30]. In the aerospace field, Al_2_O_3_ aerogels are often obtained using the organic alkoxide method because it enables the preparation of aerogels with high specific gravity and high purity, which are suitable for high-temperature service. Aging of the Al_2_O_3_ aerogel primarily involves processes, such as condensation, dehydration shrinkage, grain coarsening, and phase transformation. This is achieved by soaking the wet gel in specific solutions, such as H_2_O/EtOH and TEOS/EtOH, in certain proportions. At present, research on the influence of aging steps on the structure and properties of aerogels is limited. The drying of Al_2_O_3_ aerogel is typically performed by drying the Al_2_O_3_ wet gel. The drying methods of Al_2_O_3_ wet gel are similar to those used for other aerogels, primarily including supercritical drying and atmospheric drying. Figure 4 illustrates the different drying methods. At present, the common supercritical drying media primarily include CO_2_ and EtOH [31,32].

**Figure 5:**
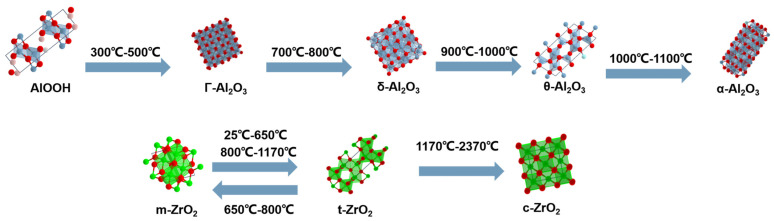
Change in crystal type of Al_2_O_3_ and ZrO_2_ aerogels with temperature.

For performance optimization and to enhance the mechanical and thermal properties of Al_2_O_3_ aerogels, researchers usually introduce reinforcing materials, such as fibers [33,34] or light-blocking agents (primarily fibers), to improve their performance. Unlike SiO_2_ aerogels, Al_2_O_3_ and ZrO_2_ aerogels undergo phase transformation during use [35], which can result in structural failure and reduced thermal insulation performance, as shown in Figure 5. Therefore, research efforts are focused on inhibiting the phase transition of Al_2_O_3_ and ZrO_2_ aerogels during use. At present, researchers often use methods such as Si doping [36] and deposition modification [37] for optimization. This aspect will be further discussed in the next chapter.

##### ZrO_2_ Aerogel and Composites for Thermal Protection

Zirconia (ZrO_2_) is an inorganic nonmetallic material known for its high-temperature resistance, wear resistance, and corrosion resistance. It exhibits low resistance at high temperatures and high resistance at low temperatures, offering excellent chemical stability and thermal stability. ZrO_2_ possesses higher chemical stability than traditional carriers and acid and alkali resistance. It has a Mohs hardness of more than 7, surpassing that of other silicate materials, making it a widely used material. In 1976, Teichner [38] and others synthesized the first ZrO_2_ aerogel, which garnered wide attention across various industries. ZrO_2_ aerogels demonstrate extraordinary properties and structural properties, becoming a prominent research topic in the field of aerogels.

ZrO_2_ aerogels possess not only the properties of general ZrO_2_, such as the easy formation of oxygen holes [39], oxidation–reduction [40], acid–base duality, and high chemical thermal stability, but also exhibit characteristics such as nanoscale structure controllability, high specific surface area, low density, high porosity, and low thermal conductivity. These properties give ZrO_2_ aerogels high application value in various fields, particularly in the potential application of thermal insulation materials, which have attracted wide attention.

Since the discovery of ZrO_2_ aerogels with their excellent properties, researchers have developed various preparation methods. Examples include gas-phase methods, such as chemical vapor synthesis and chemical vapor deposition, liquid-phase methods, such as precipitation, solvothermal, and sol–gel methods, and solid-phase methods, such as thermal decomposition and solid-phase reaction methods. Amongst these methods, the sol–gel method is a wet chemical method that offers several advantages: (1) It produces materials with uniform, fine, and narrow particles sizes; (2) the obtained materials have high purity and uniform chemical composition; and (3) the reaction conditions are mild and can be conducted at room temperature. This makes the sol–gel method the most practical approach for preparing ZrO_2_ aerogels. The following provides a brief overview of the sol–gel method. 

**Figure 6:**
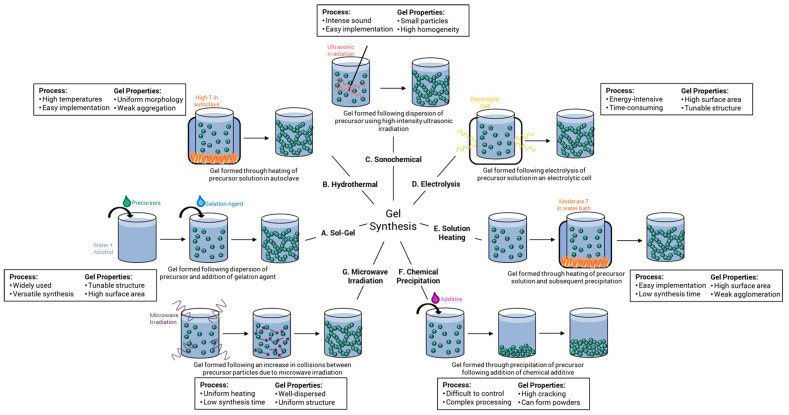
Zirconia gels can be synthesized using a variety of methods, including (**A**) sol–gel, (**B**) hydrothermal, (**C**) sonochemical, (**D**) electrolysis, (**E**) solution heating, (**F**) chemical precipitation, and (**G**) microwave irradiation methods. Each method can also be used for various aerogel systems and has different process parameters and resulting gel properties associated with it. Reproduced with permission [48].

Figure 6 describes seven different gel methods for zirconia gel: Sol–gel [41], hydrothermal treatment [42], sonochemistry [43], electrolysis [44], solution heating [45], chemical precipitation [46], and microwave radiation [47]. These gel methods can be used for various aerogel systems. Amongst these methods, the sol–gel method in wet chemical synthesis offers the following characteristics: (1) It produces materials with uniform, fine, and narrow particle sizes; (2) the obtained materials have high purity and uniform chemical composition; and (3) it allows for mild reaction conditions that can be conducted at room temperature. Therefore, the sol–gel method is an ideal and practical approach for preparing ZrO_2_ aerogels for thermal protection.

Although ZrO_2_ aerogels prepared using the aforementioned methods exhibit a high specific surface area, their high-temperature stability is poor. ZrO_2_ undergoes crystal form transformation, as shown in Figure 6, which involves a volume change and consequently leads to the destruction of its pore structure. This results in the aerogel’s limited high-temperature stability. Enhancing the high-temperature resistance of ZrO_2_ aerogels and ensuring their structural stability at elevated temperatures are important areas of development for ZrO_2_ aerogels.

#### 2.1.2. Multioxide Aerogels and Composites for Thermal Protection

As mentioned previously, Al_2_O_3_ aerogels exhibit better thermal stability at high temperatures (approximately 1000 °C) compared with SiO_2_ aerogels. Consequently, their application in specific scenarios, such as hypersonic aircraft engines, has garnered attention from researchers. However, the crystalline phase of Al_2_O_3_ undergoes changes with increasing temperatures, leading to structural failure of the aerogel and a subsequent decrease in its thermal insulation performance [49,50]. Composite oxide gels have been developed to mitigate the influence of temperature on the gel’s structure and performance. Amongst them, Al_2_O_3_-SiO_2_ and ZrO_2_-SiO_2_ aerogels and composites, obtained through various modifications of the two aerogels, have received the most extensive study.

##### Al_2_O_3_-SiO_2_ Aerogel and Composites for Thermal Protection

Pure Al_2_O_3_ aerogel is prone to sintering at temperatures exceeding 1000 °C, and the α phase transition of the crystal lattice leads to the polycondensation of the overall structure, resulting in a degradation of aerogel performance. Al_2_O_3_-SiO_2_ aerogels have been widely studied because of the ability of Si atoms to uniformly enter the center of the Al_2_O_3_ tetrahedron. This phenomenon remarkably inhibits the lattice vibration and rearrangement of Al atoms, allowing for the formation of a uniform and stable mullite phase at 1200 °C, thereby improving the thermal insulation performance of the aerogel [51]. The research in this area primarily focuses on the preparation process and performance optimization.

For the preparation process of the Al_2_O_3_-SiO_2_ aerogel, similar to single-component oxide aerogels, the process can be divided into three steps: Wet gel preparation, gel aging, and gel drying. In the case of Al_2_O_3_-SiO_2_ wet gel, the wet gel preparation involves the preparation and mixing of sols for both components. Depending on the proportions of Al_2_O_3_ and SiO_2_ aerogels, Al_2_O_3_-SiO_2_ aerogels can be categorized into Al_2_O_3_ sol systems [52,53] and SiO_2_ sol systems [54]. In Al_2_O_3_ sol systems, methanol, glacial acetic acid, and water are typically added as catalysts to form Al_2_O_3_-SiO_2_ sol gels. In SiO_2_ sol systems, ammonia water and ethanol are commonly used as catalysts to produce Al_2_O_3_-SiO_2_ sol gels. Table 2 summarizes the basic characteristics of Al_2_O_3_-SiO_2_ aerogels prepared using different wet gel preparation and gel drying processes.

**Figure 7:**
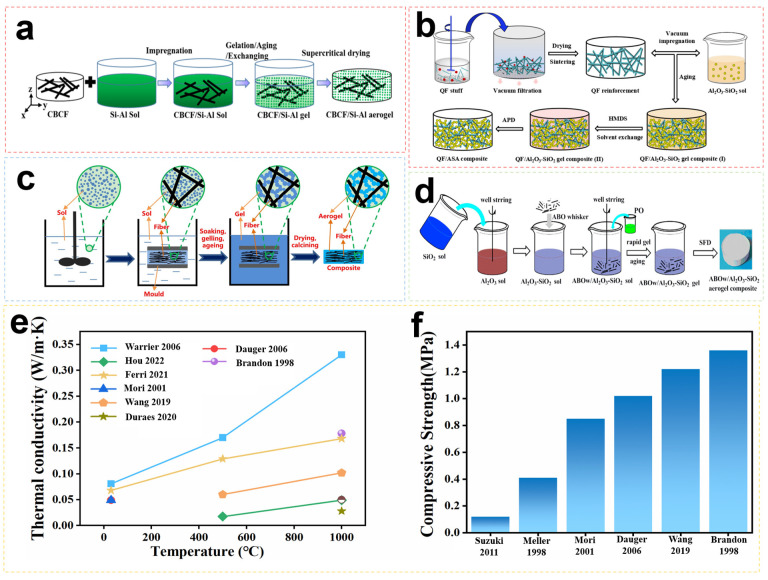
Several preparation methods for fiber-reinforced Al_2_O_3_-SiO_2_ aerogel composites: (**a**) Schematic illustration of the synthesis procedure for the CBCF/Si–Al aerogel. Reproduced with permission [63]. (**b**) Schematic representation of the sol–gel process of the QF/ASA composite. Reproduced with permission [64]. (**c**) Fabrication of the MFASs through the SIG-SCFD strategy. Reproduced with permission [65]. (**d**) Fabrication flow chart of ABOw/Al_2_O_3_–SiO_2_ aerogel composites. Reproduced with permission [66]. (**e**) Thermal conductivity of Al_2_O_3_-SiO_2_ aerogel composites in this paper. (**f**) Compressive strength of Al_2_O_3_-SiO_2_ aerogel composites in this paper.

The performance optimization of Al_2_O_3_-SiO_2_ aerogel follows a similar approach to that of single-component oxide aerogels. Both types of aerogels suffer from poor mechanical properties, which limit their application in the aerospace field. Therefore, for two-component oxide aerogels, mechanical strengthening remains a major concern for researchers. Whiskers, fibers, and particles are used as reinforcing phases in Al_2_O_3_-SiO_2_ aerogels to improve their mechanical properties. Table 3 presents the properties of Al_2_O_3_-SiO_2_ aerogels reinforced with different fibers. In terms of aerospace thermal protection, fiber composite reinforcement proves to be the most effective method for enhancing the mechanical properties of Al_2_O_3_-SiO_2_ aerogels. Figure 7 shows the schematics of Al_2_O_3_-SiO_2_ aerogel composites prepared using various methods and the performance diagrams depicting thermal conductivity and compression strength from different studies.

As a thermal protection material, the fiber-reinforced Al_2_O_3_-SiO_2_ aerogel still exhibits higher high-temperature thermal conductivity higher compared with SiO_2_ aerogel composites, highlighting the need for further reduction. In addition, the current temperature range of Al_2_O_3_-SiO_2_ aerogels is limited to approximately 1300 °C, raising the question of how to extend their performance to even higher temperatures.

##### ZrO_2_-SiO_2_ Aerogel and Composites for Thermal Protection

ZrO_2_, renowned for its high-temperature resistance and wear resistance, possesses exceptional chemical stability and thermal stability. Recent studies have explored ZrO_2_-based solid materials due to their superior chemical stability over traditional carriers, such as Al_2_O_3_ and SiO_2_. However, similar to Al_2_O_3_ aerogels, ZrO_2_ aerogel gels undergo notable phase transformations and shrinkage at high temperatures (500–1000 °C), resulting in reduced performance. Therefore, the introduction of SiO_2_ and other components is usually used to optimize their properties. Table 4 lists the properties of ZrO_2_-SiO_2_ aerogels reinforced with various fibers. Extensive research has been conducted on the preparation methods and performance optimization of ZrO_2_-SiO_2_ aerogel composites. This research primarily focuses on the selection of raw materials, the introduction of SiO_2_ additives, and the choice of reinforcing phases.

Although many strategies have been successfully applied to the preparation and modification of ZrO_2_-SiO_2_ aerogel composites, the current application temperature range is approximately 1000 °C, exhibiting inferior thermal protection compared with Al_2_O_3_-SiO_2_ aerogel gel composites. Therefore, enhancing the thermal stability of ZrO_2_-SiO_2_ aerogel composites through improved preparation processes and modification techniques represents a crucial avenue for future exploration.

### 2.2. Organic Aerogels and Composites for Thermal Protection

The study of organic aerogels began in 1987 when Pekala [81] first prepared organic monomer aerogels from resorcinol and formaldehyde under alkaline conditions using the sol–gel process and the supercritical drying method. Organic aerogels include polymer-based aerogels and biomass-based aerogels. Polymer-based aerogels are primarily used in aerospace thermal protection. These aerogels are porous network structures formed by the combination of polymer molecules and colloidal particles through hydrogen bonds or van der Waals forces [82]. Polymer-based organic aerogels utilized for aerospace thermal protection include polyimide [83] and phenolic [84].

#### 2.2.1. Polyimide Aerogels and Composites for Thermal Protection

Polyimide (PI) is a type of polymer that finds applications in engine components due to its stability, high dielectric properties, and excellent mechanical properties at high temperatures [85]. Recent research has focused on functional PI aerogels, and several PI aerogels for space exploration and electronics have been reported. Previous studies demonstrated highly flexible and even foldable PI aerogels [86]. However, these strategies primarily involve altering the chemical composition or introducing chemical crosslinkers to modify the skeleton chemistry of polyimide aerogels. Although improvements have been achieved in various properties, these traditional strategies have limitations, necessitating more effective methods to enhance the functionality of PI aerogels. Recent efforts to enhance the performance of polyimide aerogel thermal insulation materials have focused on inhibiting shrinkage and improving the temperature resistance and fibrosity of polyimide aerogels. Table 5 presents the properties of polyimide aerogel materials obtained from relevant research.

Functional additives, hybridization, and optimization of other processes are commonly employed to inhibit the shrinkage of polyimide aerogel and enhance its thermal insulation performance. The introduction of additives into PI aerogels to reduce shrinkage is achieved by utilizing their physical support, chemical crosslinking, or a combination of both. These functional additives include aerogel powder [87], silica spheres [88], and other particle-like structures. In addition, ultrafine fibers [89] and raw fiber minerals [90], such as whiskers [91] and carbon nanotubes [92], are also used as additives to improve the antishrinkage properties of PI aerogels. In addition, sheet materials, such as reduced and oxidized graphene sheets [93], are attractive as functional additives. These additives exhibit varying effects on reducing the shrinkage of PI aerogels.

**Table 5:** Properties of PI aerogels and their composites.

Raw Materials	Enhancement Phase	Density/g·cm^−3^	Thermal Conductivity/W·(m·K)^−1^	Shrinkage/%	Reference
NMP ^4^, ODA ^1^, BPDA ^2^	SiO_2_ aerogel-powders	0.020	0.028	7.5	[87]
DMAc ^3^, BPDA ^2^, ODA ^1^, TEA	SiO_2_ nanoparticles	0.080	0.020	9.0	[88]
DMAc ^3^, ODA ^1^, PAA	FHal ^8^	0.065	0.039	21.9	[90]
NMP ^4^, ODA ^1^, PMDA	SiC whisker	0.238	0.036	16.2	[94]
ODA ^1^, BPDA ^2^, PAA ^5^	CNT	0.107	0.023	6.2	[92]
PAA, LDH ^6^	GO ^9^	0.052	0.036	29	[93]
ODA ^1^, PPDA ^7^	Glass fiber	0.143–0.177	0.023–0.029	-	[89]

^1^ ODA: Triethylamine, 4,4′-oxydiphenylamine; ^2^ BPDA: Biphenyl tetraic anhydride; ^3^ DMAc: Dimethyl acetamide; ^4^ NMP: N-Methyl-2-pyrrolidinone; ^5^ PAA: Polyamide acid; ^6^ LDH: Layered double hydroxides; ^7^ PPDA: P-phenylenediamine; ^8^ FHal: Clay halloysite nanotubes; ^9^ GO: Graphene oxide.

**Figure 8:**
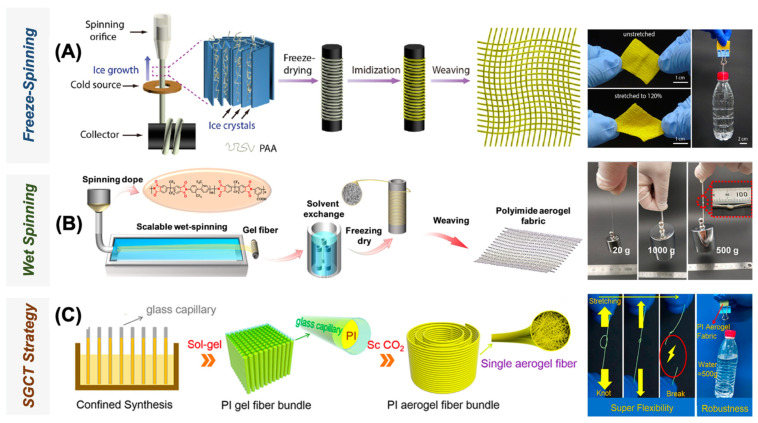
Technologies for transforming 3D aerogels into 1D aerogel fibers: (**A**) Freeze-spinning technique for fabricating stretchable textiles, (**B**) wet spinning technique for multifunctional fibers and textiles, (**C**) SGCT technique for strong and durable aerogel fiber fabrication. Reproduced with permission [98].

In the field of polyimide aerogel fibrosis, extensive research has been conducted because of the unique combination of high-temperature resistance and thermal insulation properties of PI aerogel, along with the exceptional mechanical properties of fibers. The development of PI aerogel fibers has garnered considerable attention, as they can be woven into textiles to create multifunctional fabrics, particularly suitable for applications requiring temperature regulation. This advancement holds immense potential for the next generation of smart textiles, encompassing everyday clothing, sports-wearable equipment, fire-fighting equipment, and even aerospace garments. The methods primarily employed for the preparation of PI aerogel fibers include freezing spinning [95], wet spinning [96], and capillary gel [97]. A visual representation of these specific preparation techniques is shown in Figure 8.

#### 2.2.2. Phenolic Aerogels and Composites for Thermal Protection

Phenolic resin (PFR) offers desirable characteristics, such as good mechanical properties, fire resistance, flame retardancy, chemical resistance, and weather resistance. Consequently, PFR finds wide applications in the defense and military industry, aerospace, civil construction, electronics, and electrical fields. However, traditional PFR matrix composites suffer from drawbacks, such as high density and high thermal conductivity, which limits their usage to some extent. PFR aerogels can effectively mitigate these issues by reducing material density and thermal conductivity, thereby expanding their potential applications in aerospace and other fields [99]. Recent research has focused on enhancing the ablation resistance [100] and optimizing the thermal and mechanical performance of phenolic aerogel thermal insulation [101,102] in high-temperature aerobic environments. Table 6 provides an overview of the properties of phenolic aerogel materials obtained from relevant studies.

However, pure phenolic aerogel exhibits poor resistance to high-temperature environments when it comes to ablation performance. Initially, phenolic aerogel gel was used as an antiablation material in combination with carbon fiber [99,102]. However, carbon fiber-reinforced phenolic aerogels are susceptible to oxidization in high-temperature aerobic environments, resulting in the failure of thermal protection materials. Researchers commonly adopt methods such as inorganic modification of the matrix or substituting carbon fiber with high-temperature inorganic fiber to enhance the ablation resistance of phenolic aerogel thermal insulation materials in such conditions [100,103,104]. The ablation resistance of the material can be improved by leveraging the heat resistance and oxidation resistance of inorganic components.

The microstructure of phenolic aerogels can be adjusted to enhance their thermal insulation performance, effectively reducing their thermal conductivity at room temperature and enabling the production of phenolic aerogels with varying thermal insulation properties [108]. In addition, a common approach involves incorporating phenolic aerogels into traditional inorganic aerogel gels, leveraging the nanoporous structures and intrinsic low thermal conductivity of inorganic aerogels to improve the thermal insulation performance of the composite materials [109]. However, the mechanical properties of composite materials containing phenolic aerogels may experience a certain degree of decline due to the inherent brittleness of inorganic aerogels [110].

### 2.3. Carbon Aerogels and Carbide Aerogels and Composites for Thermal Protection

Carbon aerogels and carbide aerogels offer the advantages of low density and high porosity exhibited by traditional oxide aerogels and demonstrate excellent temperature resistance in inert atmospheres. Moreover, carbon aerogels and carbide aerogels have important applications in aerospace fields, such as in the base of the return module, the nose of space shuttles, and solid rocket motors, due to their excellent high-temperature resistance.

#### 2.3.1. Carbon Aerogels and Composites for Thermal Protection

In 1987, Pekala first carbonized phenolic aerogels to obtain carbon aerogels, marking the beginning of research on carbon aerogels [81]. Under an inert atmosphere or vacuum environment, carbon aerogels exhibit high-temperature resistance of up to 2000 °C, and graphitized carbon aerogels further enhance this temperature resistance, reaching up to 3000 °C [111]. Moreover, carbon aerogels effectively inhibit high-temperature radiation heat transfer compared with traditional inorganic aerogel thermal insulation materials, resulting in a reduction of their high-temperature thermal conductivity [112]. Therefore, carbon aerogels have gained increasing attention for thermal protection applications in the aerospace field. Over the past few decades, research on carbon aerogel thermal insulation materials has primarily focused on two aspects: Improving processes and optimizing performance.

**Figure 9:**
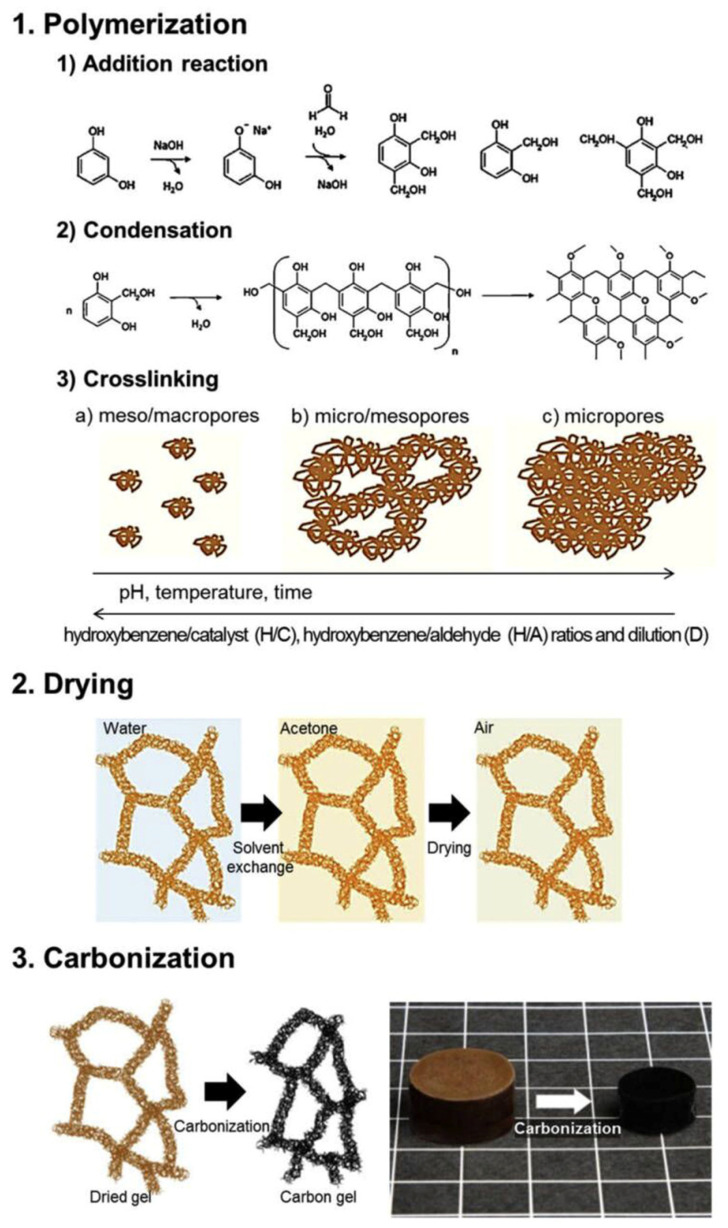
Fundamental procedure for the preparation of carbon aerogels. Reproduced with permission [115,116].

Process improvement efforts typically involve optimizing the preparation process, cross-linking the polymer, and constructing a multiscale multipenetrating network framework. Figure 9 shows the fundamental procedure for the preparation of carbon aerogels. The classification of carbon aerogels based on their precursors and their respective properties are summarized in Table 7. The supercritical drying process, commonly used for preparing oxide aerogels, shares similarities with the drying process for carbon aerogels. However, the supercritical drying process has several drawbacks, including long operation cycles, high-risk factors, high energy consumption, and high costs. These limitations greatly restrict the industrial production and application of carbon aerogels. In contrast, the atmospheric drying process is more suitable for the production of carbon aerogels. During normal pressure drying, three key factors are believed to reduce the collapse and shrinkage of the pore structure in carbon aerogels: Proper network structure strength, larger particle and pore sizes, and low surface tension [113]. The condensation reaction between polymer monomers and residual hydroxyl groups on the surface of the carbon aerogel network can lead to the formation of a polymer film. This film enriches and coats the surface of the gel’s solid network skeleton, resulting in a thicker skeleton and larger connection area between adjacent secondary particles, finally strengthening the network structure of the gel. In addition, introducing another component or multiple components to build a dual network or multinetwork skeleton structure that interpenetrates or intertwines with each other can effectively adsorb fracture energy at cracks. This mechanism prevents cracks from propagating to the macro level and effectively strengthens the gel’s network structure [114].

**Figure 10:**
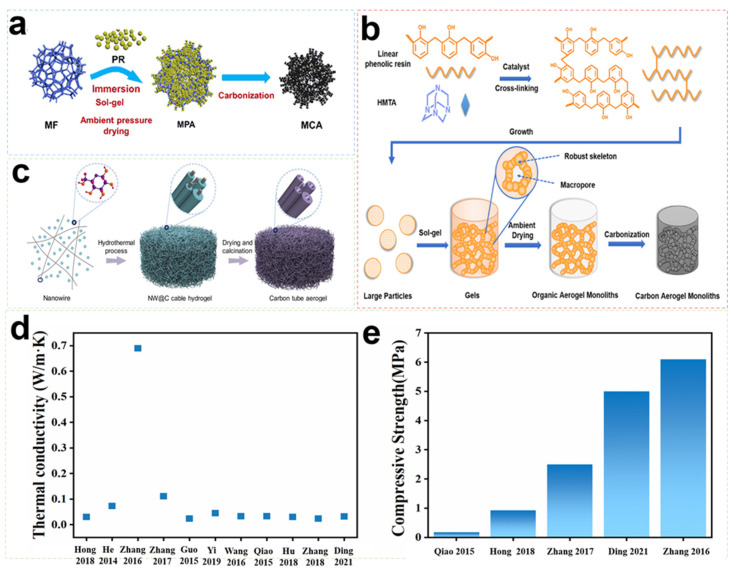
Several preparation methods of fiber-reinforced carbon aerogels: (**a**) Schematic of the fabrication process of MCA. Reproduced with permission [118]. (**b**) Schematic illustration of the preparation of OAMs and CAMs. Reproduced with permission [119]. (**c**) Schematic illustration of the bioinspired fabrication processes of CTAs. Reproduced with permission [120]. (**d**) Thermal conductivity of carbon aerogel composites in this paper. (**e**) Compressive strength of carbon aerogel composites in this paper.

For performance optimization, the addition of fibers and carbon nanomaterials to the aerogel matrix is a common method to improve their mechanical properties [117]. Figure 10 shows several preparation methods and physical properties of fiber reinforced carbon Aerogel. In terms of added fibers, the commonly used fiber tougheners in the aerospace field are primarily inorganic fibers (mullite fiber, Al_2_O_3_ fiber, carbon fiber, etc.). Amongst these inorganic fibers, carbon aerogels reinforced with fiber felts have attracted extensive attention from researchers due to their good formability and designability. Carbon nanomaterials (graphene oxide, carbon nanotubes, graphite, etc.) are also considered suitable for enhancing the toughness and thermal protection capabilities of aerogels in the aerospace field due to their unique material structure and physical and chemical properties. Amongst these carbon nanomaterials, graphene oxide has been widely studied for its excellent chemical stability and temperature resistance (up to 2000 °C). Table 8 provides information on the physical, chemical, and mechanical properties of various carbon aerogel composites.

#### 2.3.2. Carbide Aerogels and Composites for Thermal Protection

Although carbon aerogels and their composites have excellent high-temperature resistance in an inert atmosphere, reaching a maximum temperature of 3000 °C, their oxidation resistance in an air atmosphere is poor. Therefore, they need to be coated with antioxidation coatings to prevent oxidation. However, for reusable aircraft, the compactness and antioxidation performance of the antioxidation coating may decline over time due to long-term high-temperature aerodynamic heating and repeated thermal scouring. Carbide aerogel materials represent one of the most abundant branches of aerogel materials. Compared with traditional oxide aerogels, carbide aerogels offer higher-temperature resistance, reaching up to 3000 °C in an inert atmosphere, with a density of less than 0.4 g/cm^3^ and a room temperature thermal conductivity of less than 0.040 W/(m·K) [129,130]. Therefore, carbide aerogel materials have become highly promising for applications in a temperature range above 1200 °C and are widely used in aerospace and other high-temperature insulation fields.

##### SiC Aerogels and Composites for Thermal Protection

SiC aerogels have been extensively studied in extreme environments due to their stable chemical properties, good thermal shock performance, and low thermal expansion coefficient. In the past decade, research on SiC aerogel thermal insulation materials for thermal protection has focused on enhancing their mechanical properties and thermal insulation properties and developing practical preparation technologies.

To enhance the mechanical properties and thermal insulation properties of SiC aerogels, researchers have recently developed 1D SiC nanofibers as new materials. These nanofibers possess stacking faults and micro twin structures that enable them to exhibit a super strong plastic deformation ability, resulting in further improvement of the intrinsic mechanical properties of SIC aerogels [131,132]. Other scholars have prepared anisotropic and layered SiC nanowires based on template directional solidification and high-temperature heat treatment of SiC-SiO_2_ nanowire aerogels. Compared with SiC nanowire aerogel, this material demonstrates superior thermal insulation performance [133]. Unlike predominantly amorphous oxide aerogels, SiC aerogels consist of abundant crystals. Heat conduction in the SiC skeleton is primarily governed by phonon transmission, whereas phonon scattering occurs because of lattice defects, such as impurities, vacancies, lattice oxygen content, gaps, and dislocations at room temperature. These defects play a crucial role in determining the thermal conductivity of SiC aerogels [134]. Therefore, SiC aerogels exhibit good infrared shielding performance. However, their thermal conductivity is still higher than that of SiO_2_, ZrO_2_, and other oxide aerogel materials. In recent years, the addition of SiO_2_ as a sunscreen into aerogels can markedly enhance their interfacial thermal resistance and greatly reduce their thermal conductivity [135]. Two types can be distinguished on the basis of the method of introducing SiO_2_: The direct addition of nano-SiO_2_ particles into the SiC precursor to form the SiC/SiO_2_ interface as the adiabatic phase or utilizing the SiO_2_ layer formed by in situ oxidation of SiC as the adiabatic phase. The former type, which involves point contact between particles, has a limited number of new interfaces, resulting in only a slight improvement in thermal insulation performance [134]. The in situ SiO_2_ layer can effectively ‘weld’ the SiC skeleton particles together, creating a high-strength SiC/SiO_2_ composite aerogel with a core/shell structure, which exhibits excellent thermal insulation performance [136]. The performance of this composite is summarized in Table 9.

In terms of practical SiC aerogel preparation technology, the current commonly used method involves using organic/SiO_2_ composite aerogels as precursors and combining the sol–gel method with the carbothermal reduction method to produce complete blocky SiC aerogels. However, this method presents some challenges, such as a complex process, a lengthy preparation period, and the need to address the high carbothermal reduction temperature. In addition, the huge volume shrinkage during the high-temperature carbothermal reduction can lead to internal stress, making it difficult to prepare large-scale, specially shaped SiC aerogel components. Therefore, a novel approach utilizing flexible carbon fiber as a SiC support structure and growth template holds promise as a crucial direction for future research in this field. Another emerging area in practical SiC aerogel preparation involves using preceramic polymers. This method avoids dependence on the carbothermal reduction of organic/SiO_2_ composite aerogels and enables the achievement of atomic-level mixing of Si and C in the preceramic polymer precursor, resulting in a remarkable reduction in the required high-temperature heat treatment [137].

**Table 9:** Properties of SiC aerogel and its composites.

Raw Materials	Density/g·cm^−3^	Specific Surface Area/m^2^·g^−1^	Thermal Conductivity/W·(m·K)^−1^	Reference
PAN ^1^, SiO_2_	0.500	20	-	[138]
PAN ^1^, TMOS	0.320	20	-	[139]
APTES ^2^	0.29	251	-	[140]
SMP-10 ^3^	0.170	444	-	[141]
PCS-800 ^4^, KIT-6	-	942	-	[142]
Siloxane gel	0.005	78	0.026	[143]
Graphene foam	0.017	-	0.160	[144]
SiO powder, Balsa wood	-	-	0.019	[145]
SiC Nanowire	0.007	-	0.014	[133]
SiC fiber	0.039	-	0.025	[146]
Si powder, SiO_2_ powder	0.076	-	0.035	[140]

^1^ PAN: Polyacrylonitrile; ^2^ APTES: 3-aminopropyltriethoxysilane; ^3^ SMP-10:Allylhydropolycarbosilane; ^4^ PCS-800: The commercial polycarbosilane

##### Other Carbide Aerogels and Composites for Thermal Protection

With the rapid advancement of new aerospace technologies, the development of super thermal insulation materials possessing high temperature, low density, and ultralow thermal conductivity has become an important direction in the field of thermal insulation materials. Traditional aerogels exhibit high specific surface area, low density, and low thermal conductivity. However, their low strength limits their practical application. In contrast, SiOC [147], ZrC [148], ZrOC [149], and SiCNO [150] aerogels offer higher strength and superior high-temperature stability compared with SiO_2_ aerogels. This is attributed to the partial replacement of oxygen atoms in traditional oxides, such as SiO_2_ and ZrO_2_, with carbon atoms in the tetravalent state. This substitution effectively increases the density of chemical bonds and forms a robust molecular network structure, resulting in excellent thermal stability and mechanical properties in the synthesized ternary carbide aerogels. These advantages overcome the shortcomings associated with low oxide strength and the susceptibility of binary carbides to oxidation at high temperatures. Consequently, these new C5 aerogels are expected to become the next generation of high-performance aerogel insulation materials suitable for aerospace thermal protection systems.

## 3. Application of Aerogels for Thermal Protection in the Aerospace Field

Since the early 1990s, the ASPEN Company of the United States, with the support of NASA, has been developing fiber-reinforced aerogel composite technology and conducting research on the application of nanoporous thermal insulation composites in various aerospace applications. These include hypersonic aircraft reentry thermal protection systems, cryogenic tanks and valve pipe insulation systems for liquid rocket fuel, noise reduction, and thermal insulation systems for warships and aircraft engines’ thermal insulation systems. In recent years, aerogels and their composites have found diverse applications in the aerospace field.

As early as 1997, SiO_2_ aerogel materials were used as thermal insulation materials in the aerospace field in the United States. NASA filled a 25–32 mm SiO_2_ aerogel (with a thermal conductivity of 0.0163 W/(m·K)^−1^) with thermal insulation properties into the structural plate of the electronic element incubator (WEB) of the Mars probe ‘Traveler’. This application aimed to safeguard the main battery pack of the probe’s alpha particle X-ray spectrometer from the impact of extremely low temperatures [151]. Building on the success of using aerogel in the Mars mission, NASA used a 0.4% graphite-doped SiO_2_ aerogel as the thermal insulation material for electronic components in the Mars rovers ‘Spirit and Opportunity’ in 2003. This further reduced the negative impact of thermal radiation and ensured the normal operation of the detector within a temperature range of −20–90 °C [152]. In 2011, during the launch of NASA’s Curiosity Mars probe, graphite-doped SiO_2_ aerogel was utilized as the thermal insulation material on the chassis of the Mars rover. It was also used to provide heat insulation for the multimission radioisotope thermoelectric generator heat exchanger, which powers the system [153]. In addition, the spacesuit used requires excellent thermal protection in the Martian space environment to ensure astronauts’ safe extravehicular activities on Mars. With the support of NASA Johnson Space Centre, the Aspen Company has developed a fiber-reinforced silica aerogel flexible composite fiber material. Its thermal conductivity in the Martian low vacuum environment is 0.005 W/(m·K)^−1^, which is only one-fifth of that of multilayer insulation structures [154]. In NASA’s deep-space exploration activities, such as Mars exploration, PI nanoaerogels are applied to the flexible thermal protection system of the Hypersonic Inflatable Aerodynamic Decelerator to provide adiabatic insulation [155].

In 2000, the NASA Ames Research Centre developed the ceramic fiber aerogel composite heat shield, which was applied as the thermal insulation material for the space shuttle, demonstrating a thermal insulation performance 10 to 100 times higher than the original shield. This new type of heat shield can also be used in the thermal insulation layer of future reusable spacecraft and fuel tanks [156]. In 2008, NASA applied SiO_2_ aerogel material on the outer wall of the liquid hydrogen storage tank of a launch vehicle, ensuring the fuel tank’s normal operation at low temperatures and greatly reducing the weight of the space shuttle [157]. Aerogel materials have also found applications in military aircraft, particularly for thermal insulation protection of cabin bulkheads and important instruments in passenger aircraft. They are primarily used in aircraft in the United States and Britain. For example, both the MKV-22 ‘Osprey’ tiltrotor cabin wall thermal insulation system and the infrared system of the United States utilize aerogels. Similarly, aerogel materials were used in the cockpit thermal insulation wall of the modified British ‘Jaguar’ fighter.

## 4. Conclusions

The development of aerogels for thermal protection relies on advanced aerogel materials and multifunctional integrated thermal protection structures. Expanding the capabilities of existing thermal protection aerogel materials to withstand extreme service environments, exploring new material systems for thermal protection, and innovating the design concept of integrated thermal protection structures are crucial for advancing aerospace vehicle technology. Although considerable achievements and breakthroughs have been made in the research and application of aerogel thermal insulation materials, numerous challenges still need to be addressed. The current difficulties and possible future development directions primarily focus on the following aspects.

The excellent properties of aerogels, such as their lightweight nature and thermal insulation, are closely related to their unique microstructure. Adjusting the gel’s structure primarily depends on key preparation processes, such as sol–gel, aging, and drying. Building upon existing research, further investigations into the relationship between the preparation, structure, performance, and application of aerogels will lead to the development of higher-performance aerogels, thereby advancing the research and application of aerogel materials in the aerospace field.

For oxide aerogels, on the one hand, it is necessary to develop a new generation of aerogel materials with a high melting point and low thermal conductivity; for example, zirconium-based compounds, hafnium-based compounds, etc. On the other hand, it is necessary to develop sintering problems that suppress temperature-resistant components. To solve the current technical difficulties. For organic aerogels, phenolic aerogel and polyimide aerogel are still key research directions. It is necessary to further improve their anti-ablation ability in the use process and study their dimensional stability. On the other hand, it is necessary to search for low-cost organic raw materials to achieve large-scale preparation and engineering applications. For carbon aerogels, finding the balance between oxidation resistance and mechanical and thermal properties is the focus of future research. In addition, reducing production costs by shortening the preparation cycle is also one of the challenges that need to be overcome.

The rapid development of aerospace technology has introduced new requirements for thermal protection systems with high performance, including high-temperature resistance, lightweight characteristics, and high transmission capabilities. Through the structural design and performance optimization of various aerogels, key technologies such as the development of high-temperature-resistant aerogels, ultralow density aerogels, and wave transparent aerogel gels have already been achieved, initially meeting the needs of various aircraft. However, as the future service environment becomes more complex and demanding, the comprehensive performance of aerogel materials, such as temperature resistance, heat insulation, load-bearing capacity, wave transmission, and stealth capabilities, needs to be further improved to meet the evolving requirements.

## Figures and Tables

**Table 1:** Physical properties and mechanical parameters of several inorganic fibers [19].

Type of Inorganic Fiber	Density/g·cm^−1^	Tensile Strength/MPa	Service Temperature/°C
Quartz fiber	2.20	6000	1200
Glass fiber	2.48	4800	450
Aluminum silicate fiber	2.20	800	1260
Mullite fiber	3.17	1400	1400
Alumina fiber	3.70	2080	1600

**Table 2:** Basic characteristics of Al_2_O_3_-SiO_2_ aerogel.

Raw Materials	Drying Method	Drying Medium and Operating Conditions	Density/g·cm^−3^	Specific Surface Area/m^2^·g^−1^	Shrinkage at High Temperature/%	Thermal Conductivity/W·(m·K)^−1^	Reference
AIP, TEOS	Supercritical drying	EtOH (10 MPa, 300 °C)	-	99 (1300 °C)	14 (1300 °C)	-	[55]
ASB, TEOS	Supercritical drying	EtOH (10 MPa, 300 °C)	0.249	120.6 (1200 °C)	-	-	[56]
Al (NO_3_)_3_, TEOS	Atmospheric drying	30 °C	0.5	304.2 (1000 °C)	-	-	[57]
AlCl_3_, TEOS	Supercritical drying	EtOH (10 MPa, 300 °C)	0.053	120 (1200 °C)	40 (1200 °C)	-	[58]
AlCl_3_, TEOS	Supercritical drying	EtOH (10 MPa, 300 °C)	-	124.2 (1200 °C)	-	0.0275	[59]
ASB, TMEO	Supercritical drying	EtOH (10 MPa, 300 °C)	-	72 (1200 °C)	38 (1200 °C)	-	[60]
AlCl_3_, TEOS	Supercritical drying	EtOH (10 MPa, 260 °C)	-	234 (1000 °C)	-	0.05	[61]
γ-AlOOH, TMOS	Supercritical drying	EtOH (10 MPa, 300 °C)	0.146	79 (1200 °C)	2.5 (1300 °C)	-	[62]

**Table 3:** Properties of Al_2_O_3_-SiO_2_ aerogel composites.

Enhancement Phase	Density/g·cm^−3^	Room Temperature Thermal Conductivity/W·(m·K)^−1^	High Temperature Thermal Conductivity W·(m·K)^−1^	Compressive Strength/MPa	Reference
MF ^1^ (SiC)	-	-	0.049 (1000 °C)	-	[32]
MF ^1^	0.36	-	0.082 (1200 °C)	0.12	[65]
MF ^1^ (TiO_2_)	0.23	0.068	0.168 (1050 °C)		[67]
CNT	0.23		0.178 (1000 °C)	1.36	[68]
ABO_W_ (30%) ^2^	0.35	0.049	-	1.02	[66]
Kevlar (R)	0.12	0.028	-	-	[69]
Quartz fiber	0.36	0.049	-	0.85	[64]
Al_2_O_3_-SiO_2_ fiber	0.33	0.050	-	0.41	[70]
ZrO_2_ fiber	0.59	0.049	0.102 (1000 °C)	1.22	[36]
Carbon fiber	0.37	0.081	0.330 (1000 °C)	-	[63]

^1^ MF: Mullite fibers; ^2^ ABO_W_: Aluminum borate whisker.

**Table 4:** Properties of ZrO_2_-SiO_2_ aerogel and its composites.

Raw Materials	Enhancement Phase	Density/g·cm^−3^	Specific Surface Area/m^2^·g^−1^	Room Temperature Thermal Conductivity/W·(m·K)^−1^	Compressive Strength/MPa	Reference
ZrOCl_2_, Na_2_SiO_3_	-	0.136	383 (1000 °C)	0.026	-	[71]
ZrOCl_2_, TEOS	-	0.270	228 (1000 °C)	-	-	[72]
ZrOCl_2_, TEOS	-	0.290	-	0.027	-	[73]
ZBO ^1^, TEOS	-	-	172 (1000 °C)	-	-	[74]
ZrO (NO_3_)_2_, TEOS	-	0.202	-	-	-	[75]
PAZ ^2^, TEOS	-	0.144	214 (1000 °C)	-	-	[76]
ZrOCl_2_, TEOS	PMF	0.450	-	0.052	1.05	[77]
ZrOCl_2_, TEOS	MF	0.225	-	0.027	0.438	[78]
ZrOCl_2_, TEOS	ZrO_2_ Fiber	0.302	-	0.034	0.170	[79]
ZrOCl_2_, TEOS	ZrO_2_ Fiber	0.290	-	0.029	0.530	[80]

^1^ ZBO: Zirconium (IV) butoxide; ^2^ PAZ: Polyacetylacetonatozirconium.

**Table 6:** Properties of PI aerogel and its composites.

Raw Materials	Enhancement Phase	Density/g·cm^−3^	Thermal Conductivity/W·(m·K)^−1^	Linear Ablation/mm s^−1^	Reference
PR ^1^, HMTA ^2^	Carbon fiber	0.270–0.370	0.093–0.230	0.029 (1.5 MW/m^2^ 33 s)	[102]
PR ^1^, ZrB_2_, SiB_6_	Quartz fiber felt	0.348	-	0.017	[103]
PR ^1^, HMTA ^2^, MTMS ^3^, DMDES ^4^	Carbon fiber felt	0.30–0.35	0.068	0.019 (1.5 MW/m^2^ 300 s)	[99]
PR ^1^, MTMS ^3^, DMDES ^4^, APTES ^5^	Quartz/carbon hybrid fiber	0.310–0.350	0.050–0.063	0.058 (3.62 MW/m^2^ 300 s)	[104]
PR ^1^, APTES ^5^, HMTA ^2^	Quartz fiber	0.200	0.048	0.010 (1.5 MW/m^2^ 180 s)	[100]
PR ^1^, HMTA ^2^	Glass fiber wool	0.036–0.140	0.031–0.037	-	[105]
PF, HMTA ^2^	-	~0.112	0.021	-	[106]
PR ^1^, HMTA ^2^	Quartz felt	~0.016	~0.030	0.003 (1.5 MW/m^2^ 300 s)	[107]

^1^ PR: Phenolic resin; ^2^ HMTA: Hexamethylenetetramine; ^3^ MTMS: Methyltrimethoxysilane; ^4^ DMDES: Dimethyldiethoxysilane; ^5^ APTES: 3-Aminopropyltriethoxysilane.

**Table 7:** Classification and properties of carbon aerogels based on precursors. Reproduced with permission [115].

Types	Precursors	Properties
synthetic polymer-based carbon aerogel	✓ aromatics (phenol, cresol, phloroglucinol) and aldehydes (furfural, formaldehyde)	Textural properties controllable by synthesis conditions Uniform morphology through bottom-up process
	✓ polymers (poly (vinyl alcohol), poly (vinyl chloride), polyimide)	Applicable to large-scale production
Graphitic materials-based carbon aerogel	✓ carbon nanotube (CNT), graphene, carbide, carbonitride	Crosslinked each other through van der Waals interactions Promising candidates as electrically conductive materials Carbonization process is skippable
Biomass-based carbon aerogel	✓ hydrated biomass (watermelon, cucumber, aloe, celery, pumpkin)	Not required for the gelation process
	✓ highly porous biomass (cotton, cattail, cane)	Porous structure obtained via sublimation of water in hydrated biomass Inexpensive, abundant, and eco-friendly

**Table 8:** Properties of carbon aerogel and its composites.

Raw Materials	Enhancement Phase	Density/g·cm^−3^	Thermal Conductivity/W·(m·K)^−1^	Compressive Strength/MPa	Reference
P ^1^, HMTA ^2^	UCF ^10^	0.16	0.030	0.93	[121]
R ^3^, F ^4^	PAN fiber	0.17	0.073	-	[122]
R ^3^, F-F ^5^	PAN fiber	0.68	0.690	6.10	[123]
P ^1^, MF ^6^	-	0.12	0.111	2.50	[118]
PPA ^7^, GO	-	0.11	0.023	-	[124]
DMF ^8^, PPA ^7^, GO	-	-	0.045	-	[125]
GO	Quartz fiber	0.07	0.033	-	[126]
GO	-	0.13	0.033	0.18	[127]
MWCNTs	-	-	0.030	-	[128]
Te NWs ^9^, glucose	-	-	0.023	-	[120]
P ^1^, H	-	0.07	0.032	5.00	[119]

^1^ P: Phenolic resin; ^2^ HMTA: Hexamethylenetetramine; ^3^ R: Resorcinol; ^4^ F: Formaldehyde; ^5^ F-F: Furfural; ^6^ MF: Melamine foam; ^7^ PPA: Paraphenylene diamine; ^8^ DMF: N, N-Dimethylformamide; ^9^ Te NWs: Te nanowire; ^10^ UCF: Ultralight carbon fiber.

## Data Availability

No new data were created.
